# The Value Added of Incorporating Qualitative Approaches into Malaria Surveillance, Monitoring, and Evaluation

**DOI:** 10.4269/ajtmh.22-0191

**Published:** 2022-12-12

**Authors:** Lwendo Davis, Debra Prosnitz, Yazoume Ye

**Affiliations:** ^1^ ICF, Rockville, Maryland;; ^2^ ICF, Berkeley, California;; ^3^ CDC Foundation, Atlanta, Georgia

## Abstract

Progress toward malaria elimination and improvements in the performance of national malaria control programs (NMCPs) have stalled in recent years. The current COVID-19 pandemic further threatens building on previous gains. Surveillance, monitoring, and evaluation (SME) are critical for the continued success of NMCPs because they provide the information necessary for effective program planning and management. Interventions aimed at strengthening NMCPs focus on both the target population and the program provider. Qualitative approaches are often used to understand the target population and barriers to intervention success. Although there is growing emphasis on qualitative approaches in provider-focused SME, metrics of success tend to focus on quantitative measures. The integration of qualitative approaches offers added value because they provide additional data points to facilitate the understanding of barriers that impede sustaining the gains made from provider-focused capacity-building efforts. Qualitative approaches focus on understanding program implementation and interventions, but the systematic integration of qualitative data is limited. Qualitative approaches provide avenues to strengthen SME efforts, can lead to subsequent improvement for NMCPs, and fuel progress toward malaria elimination.

## INTRODUCTION

There has been tremendous progress made on the prevention and elimination of malaria, specifically between 2000 and 2016 but, since then, progress has stalled. Progress has slowed due, in part, to the unmet need in global funding, and it is further compounded by the global COVID-19 pandemic and its negative impact on communities, the health workforce, supply chains, and overall health systems.^1^ For example, due to the disruption in services as a result of the COVID-19 pandemic, in 2020 there were 14 million more malaria cases and 47,000 more deaths than in 2019.^2^ To get back on track and continue to make progress and build on prior gains made, it is imperative to understand the country-specific context and the environments and socioeconomic dynamics in the locations where malaria programs are deployed.^3^ In 2020 and 2021, multilateral and bilateral partners updated and developed new strategies in the fight against malaria. Commonalities across these strategies include country ownership and leadership, engagement of communities, data-driven approaches, the development and introduction of new tools, and funding adequacy.^2^ However, the fight against malaria cannot succeed without strengthening surveillance, monitoring, and evaluation (SME). SME efforts include the ongoing and systematic tracking of health issues, to aid in understanding what is being done to address the issues, what gains have been made, and which issues are still persistent.^4^ In general, SME efforts are aimed at increasing the effectiveness of programs based on program data and evaluation, providing guidance on decision making around resource allocation and use, and attaining national and global goals to control and eliminate malaria.^4^ SME efforts foster streamlining processes set forth in strategies such as the Global Technical Strategy for Malaria 2016–2030 and the High Burden to High Impact strategy, which rely on pillars such as strategic information to drive impact and improve guidance, policies, and strategies.^2^

The objective of this scoping study was to gain an understanding of what approaches are promoted to improve SME efforts and the extent to which guidance on qualitative methods, approaches, and resources is provided. Based on the study, this paper elucidates how qualitative approaches can provide more insight into the challenges affecting the performance of malaria SME. Throughout this paper, we use the terminology “qualitative approaches” broadly to include both the collection of qualitative malaria data and the use of qualitative research methods to assess and improve routine malaria surveillance. Qualitative approaches focus on collecting and analyzing nonnumerical data generally obtained through observations, interviews, discussions, and documents. Qualitative data are critical for providing insights into why we are seeing trends and what can be done about them. Although SME efforts provide an understanding that progress toward meeting malaria goals has stalled, there is limited understanding about why and what to do about it, and qualitative approaches can provide this much-needed information.

Global malaria activities focus on sustaining, improving, and expanding efforts to control the disease. Interventions aimed at improving national malaria control programs (NMCPs) focus on both the target population and the SME staff and health care provider. Efforts focused on the population at risk have integrated qualitative approaches and methods, namely through efforts aimed at behavioral change. Several studies use qualitative approaches to understand barriers around intervention uptake or reasons why specific interventions were successful, obtain an understanding of the level of knowledge about a particular malaria intervention, and obtain lessons learned from the implementation of malaria surveillance strategies.^5^^–^^8^ Thus, qualitative approaches have primarily been applied to understand program implementation and interventions; however, systematic integration of qualitative methods and approaches to improve challenges that surround the provider is limited. Generally, the focus on the SME staff or health care professional who collects health care data is around providing the information, resources, and technical assistance that are thought to be beneficial for SME. These can come in the form of training, workshops, or the provision of data collection and analysis tools. Qualitative approaches can provide insights into persistent challenges and barriers that providers face as they carry out SME and corrective actions based on those efforts.

In this paper, we examine the current ways in which qualitative approaches are currently incorporated into SME, the gaps that currently exist, and the areas for improvement. We argue that an integrated qualitative approach will result in increased evidence of improved quality of SME for malaria programs, documentation of persistent challenges that are likely to impede effective surveillance as an intervention, and a more balanced, comprehensive approach to SME.

## MATERIALS AND METHODS

The authors of the manuscript are affiliated with the Measure Malaria project, which is a United States Agency for International Development–funded project with the mandate of strengthening health information systems in low-resource settings. Thus, this study was conducted in countries where there is ongoing support to strengthen malaria SME through the Measure Malaria project. We leveraged our connections in these countries to access and interview field-based staff working on malaria SME efforts.

### Interviews with partners.

The research and data collection for this study began in March 2020 and were ongoing through the submission of the first draft of this manuscript in March 2022. We began with consultative discussions with country partners implementing malaria SME activities across several countries. Discussions were conducted by phone with 10 individuals, including SME advisors from various President’s Malaria Initiative Measure Malaria–operating countries. These discussions identified key SME resources and approaches that NMCPs were implementing and provided anecdotal information on the extent to which qualitative methods and approaches were integrated into SME efforts. Additional insights were also enlisted during conferences, meetings, and workshops in which the topic of this study was presented.

### Document review.

**Figure 1.**
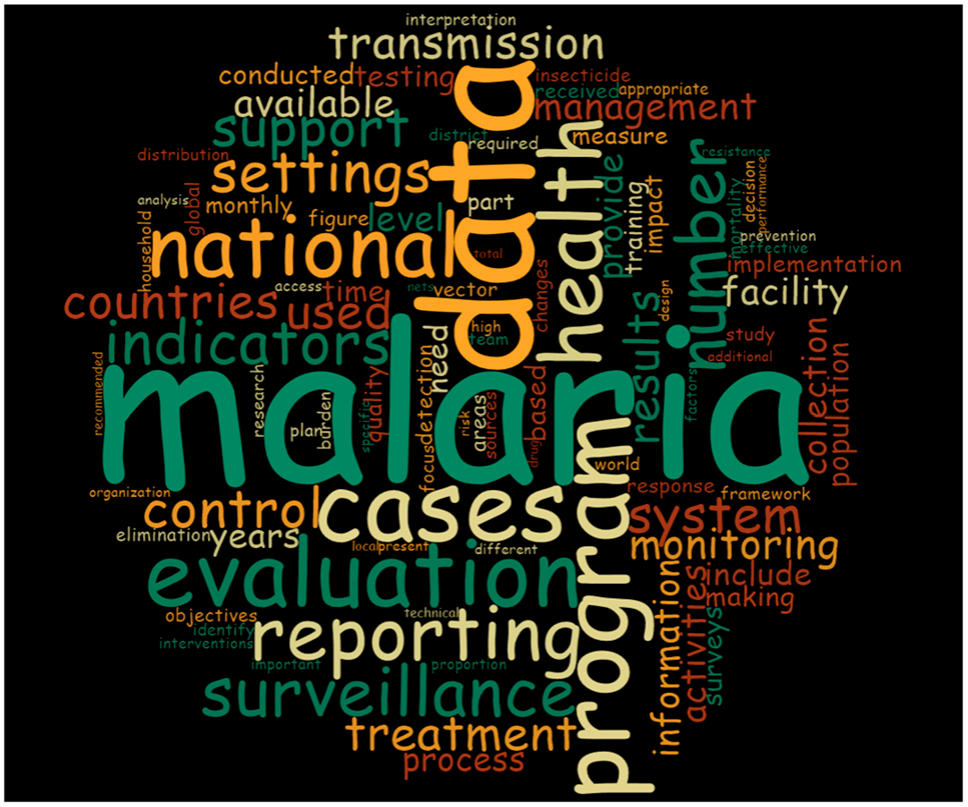
NVivo word cloud.

A targeted and purposive search was conducted to identify articles, tools, guidance, and manuals related to SME of NMCPs. The targeted search included a combination of the following terms and phrases: “surveillance, monitoring, & evaluation,” “malaria,” “qualitative approaches,” and “qualitative methods.” The initial search was conducted to obtain a general sense of SME for NMCPs, the approaches promoted, and the tools and resources implemented. Google Scholar was the primary search engine used, and several resources were also obtained from the MEASURE Evaluation Resources page.^9^ Tools, manuals, and guidance documents were reviewed and selected resources were imported into NVivo Plus for analysis. Exploratory features and NVivo Plus tools were used to analyze the documents and assess the extent to which qualitative methods or approaches were integrated. Auto-coding features were used to explore the content of documents. Word clouds and word frequency searches were used to explore the frequency of occurrence and patterns (see Figure 1: NVivo word cloud for the reviewed resources).

## RESULTS

### Value added from qualitative approaches.

The value of integrating multiple methods and approaches is well acknowledged.^10^^–^^13^ As such, the benefits of mixed-methods approaches are accepted, specifically because qualitative data focus on aspects that cannot be measured numerically and can provide insights into complex phenomenon or reasons behind specific successes or challenges. However, the application and use of qualitative approaches and tools are lacking in malaria SME, and gaps remain in the capacity to conduct and triangulate qualitative research. The integration of qualitative approaches can also help sustain and promote gains, because qualitative metrics and data capture perceptions of changes achieved and allow for the necessary nuance and depth needed to understand why gains have stalled and how best to continue to improve SME efforts.^14^^–^^16^ Table 1 summarizes the objectives of SME, the challenges associated with trying to strengthen SME efforts, and the added value of integrating qualitative approaches in these efforts.

**Table 1:** Value added in a qualitative approach to SME

	Surveillance	Monitoring	Evaluation
Objective	Improves information collected to be used for action.	Allows program stakeholders to understand whether the program is achieving objectives and effectively using resources.	Determines the impact and significance of a program or intervention.
Challenges	Limited systematic and ongoing collection of information; inconsistent integration into other routine systems; and limitations in appropriate analysis, interpretation, and use of information for action.	Limited resources to collect information on a routine basis, challenges with completeness and quality of routine data, and limited understanding of how to collect and analyze qualitative data and triangulate findings with quantitative data.	Limited understanding of the implementation of the appropriate data collection methods and analytical approaches; difficulties in interpreting findings to ascertain the impact.
Value added in qualitative approach	Provides a deeper understanding of how to improve the information collected, strengthen resource human capacity, and facilitate sustained action based on that information. Can identify bottlenecks associated with reporting into routine systems and ways to mitigate those limitations.	Provides insights on programmatic achievements and resource utilization, and facilitates the supplementation of monitoring efforts. Can provide an understanding of the bottlenecks or challenges in collecting high-quality data.	Provides alternative data collection methods that may be more applicable for certain evaluation questions. Complements quantitative evaluation findings and the interpretation of results to help understand impact.
	Provides understanding of barriers to implementing SME findings and corrective actions and facilitates the triangulation of qualitative data through techniques such as observations, interviews, and use of participatory tools.		

SME = surveillance, monitoring, and evaluation.

### Tools, resources, and approaches.

Through a review of the literature and discussions with SME experts, several SME-related tools and resources were identified. Many of the tools, resources, and guidance documents encourage and promote the use of qualitative methods for data collection. However, the provision of guidance on how to collect, analyze, and interpret the data within these resources is limited.^3^

Table 2 highlights some of these tools and resources as well as the objectives and methodological approaches of each resource, and specifies how qualitative elements are promoted or integrated.

**Table 2:** Summary of qualitative elements in SME-related tools

Name	Objective	Methods/approach	Qualitative elements
SME of Malaria Programs: Online Course^17^	To provide a comprehensive introduction to SME of malaria programs	Educational tool that provides a wide range of modules around SME	Notes the utility and types of qualitative data collection methods (interviews, focus group discussion, and direct observation) useful for SME efforts
Malaria Routine Data Quality Assessment Tool: A Checklist to Assess the Quality of Malaria Program Data^18^	To support targeted, rapid data quality assessment forced on malaria data for use in routine data quality monitoring as part of regular supervision efforts	Excel-based, checklist format tool that provides an evaluation of timeliness and completeness and reporting accuracy, cross-checks data across various data sources, checks for consistency in the reported data, and provides an overall systems assessment	Allows for comments, which can be used to collect qualitative information
Malaria Matchbox Tool^3^	To provide guidance on how to identify risk factors and barriers to equitable and integrated people-centered malaria programs	Modular structure to provide resources, guidance, and data collection tools	Provides comprehensive qualitative data collection approaches, including reference resources, sample questions, and proposed analytical approach
PRISM Toolkit^19^	To assess the reliability and timeliness of routine health information systems, making evidence-based decisions and identifying gaps	Series of questionnaire-based tools employing varying data collection approaches; data collected are quantified or categorized to develop scores or rankings	Promotes the use of various qualitative data collection techniques, document reviews, key informant interviews, and observations
World Health Organization: Practical Manual for Malaria Programme Review and Malaria Strategic Plan Midterm Review^20^	To provide guidance and clear operational steps on how to conduct malaria program reviews and midterm reviews	Provides guidance on which data should be collected and how these data should be compiled and analyzed	Provides guidance on how to implement or conduct various qualitative data collection efforts, desk reviews, observations, and site visits
WHO Malaria Surveillance Assessment Toolkit^21^	To provide a systematic approach to measuring the performance of malaria surveillance systems and identifying and evaluating the determinants of that performance	Builds on the PRISM tools and includes a range of tools to facilitate monitoring the quality of malaria surveillance and understanding its strengths and weaknesses	Promotes the use of qualitative methods and provides question banks
Facilitating SME in Malaria-Endemic Countries: A Compendium for National Malaria Programs^4^	To serve as a resource for national malaria program personnel who need to learn and apply SME skills	Information, resources, and guidance organized by chapter with relevant information	Highlights the types of qualitative data that can be used for SME purposes, discusses how qualitative data can be used, and provides relevant resources

PRISM = Performance of Routine Information System Management; SME = surveillance, monitoring, and evaluation.

As noted in Table 2, many of the tools highlight an aspect of qualitative data collection but provide limited guidance on data use or how to triangulate findings from qualitative data collection efforts with quantitative data. Further, in several tools, the qualitative data collected are subsequently quantified, and the analysis is limited to those quantified data. Although a qualitative data collection approach is applied in some resources, SME advisors reported that the richness and nuance that emerge from the qualitative data are often not documented or captured, and thus less likely to be used to inform decision making. The recently published Malaria Matchbox Tool (2021) is one of the few resources that takes the promotion of qualitative methods further by providing qualitative data collection tools and guidance on how to analyze and use the information collected.^3^ The most common noted forms of qualitative data collection promoted include desk reviews and interviews, but there are limited examples of guided questions or guides. Further, guidance on how to identify the number of interviews that should be conducted and how to conduct analysis is also limited. Guides such as the WHO Malaria Surveillance Assessment Toolkit include recommendations on who to interview, but guidance on how to deal with and interpret data from a large number of interview participants is limited. Thus, SME staff reported feeling overwhelmed by the amount of qualitative data that is collected as well as having limited guidance on how to analyze and systematically incorporate and interpret the findings.

### Challenges and gaps.

Findings from the literature review highlight several gaps that can be best addressed through the use of qualitative methods and approaches. Even when qualitative data are available, the integration of these data with quantitative data to promote programmatic decisions is limited.^22^ Studies have also identified the lack of positive behavioral change as a major bottleneck to successful program implementation.^23^ As noted, there is a gap in the application of qualitative approaches to improve malaria SME, especially regarding the performance of providers. A primary guiding framework for provider-related work is the health information cycle.^24^ The health information cycle includes the following stages: recording; reporting; analysis; presentation; interpretation and evaluation; and dissemination and use. At each stage of the information cycle, qualitative approaches can provide valuable enhancements. These improvements are 2-fold, in that qualitative data can be collected as part of the stage in the information cycle (i.e., taking notes to document additional insights as part of the recoding process) or to help improve the data collection process or interpretation (i.e., using qualitative data collection methods such as observations to understand challenges providers face in recoding information). In general, qualitative approaches can improve the stages within the information cycle by identifying challenges and bottlenecks, providing additional data points, contextualizing information, and diversifying the way information is presented to better facilitate data use.

## DISCUSSION

The objective of this study was to gain an understanding of the approaches that are promoted to improve SME efforts and the extent to which guidance on qualitative methods, approaches, and resources is provided. Further, the study highlighted how qualitative approaches can provide more insights on the challenges affecting the performance of malaria SME. Not only should qualitative data be integrated into routine program data, they should also be incorporated to understand challenges in SME and persistent barriers that are limiting the realization of gains. Core to the concept of SME is the use of data for decision making. The combination of multiple, and at times divergent, data sources makes for a more robust evidence base from which decisions can be made. Qualitative data are not only a key foundation of this evidence base, but qualitative analytical techniques and approaches can facilitate the analysis of these data to better inform decision making.

Much of the existing SME guidance focuses on the reporting and health information cycle, and what—based on the data collected in this cycle—needs to be analyzed and interpreted. However, the ways in which qualitative approaches can be best integrated are not systematically documented, although it is well acknowledged that combining multiple data sources facilitates considering plausible contributions. There has been increasing focus on qualitative indicators in helping unpack details and idiosyncrasies of local contexts and processes.^25^ Qualitative indicators capture perceptions of changes achieved and allow for the necessary nuance and depth in monitoring and evaluation.

It is important to note that there are challenges in integrating qualitative approaches into existing SME efforts. Qualitative approaches can be time consuming, and there is limited guidance on how to systematically collect, analyze, and use the data to improve SME for NMCPs. Despite the challenges, an integrated qualitative approach will result in increased evidence of improved quality of SME for malaria programs, identification of new gaps or approaches to enhance quality, and a more balanced, well-rounded, and comprehensive approach to SME. Outside of malaria SME, existing tools and resources can be integrated to improve efforts. Such tools can serve as a resource to add modules on qualitative data collection, analysis, and triangulation to malaria SME training. Further, it is important to note that qualitative data collection is intended to be complementary and not in competition with quantitative data. For example, it would be beneficial to identify outstanding questions from quantitative data collection efforts to better target qualitative data collection efforts.

### Limitations.

The insights presented were based on discussions with a small group of individuals and a targeted review of the literature, which were all restricted to the field of malaria. Thus, the insights we derived are from a narrow viewpoint. For example, there might be examples in other areas in which there is a stronger integration of qualitative methods and approaches. However, this paper was not intended to be exhaustive. Rather, the aim was to provide readers with the opportunity to consider the value added in incorporating qualitative approaches into malaria SME.

## CONCLUSION

Although there has been an increase in the promotion of qualitative approaches, especially in recent tools and guidance, integrating qualitative approaches into the implementation of malaria SME is still limited. Qualitative approaches are often implemented to assess successes and challenges and identify potential areas of improvement. Qualitative data allow us to observe trends and patterns in the data and the indicators that are tracked. This information helps provide deeper insights into the patterns observed and considers the sociocontextual factors. These benefits are especially useful in trying to identify nuances that are impeding optimal success. Qualitative approaches are best suited to identifying persistent gaps and implementing corrective action. Although the use of qualitative data collection methods is often promoted, guidance on how to collect, analyze, and use the information is limited.
